# Effects of cognitive-motor dual-task walking exercise versus walking-only exercise on cognitive function and patient-centred outcomes in older adults with subjective brain fog: a randomised controlled trial

**DOI:** 10.1136/bmjopen-2026-121892

**Published:** 2026-10-20

**Authors:** Emre Dansuk, Burak Menek

**Affiliations:** 1 Department of Physiotherapy and Rehabilitation, Faculty of Health Sciences, Istanbul Medipol University, Istanbul, Turkey; 2 Department of Physiotherapy and Rehabilitation, Faculty of Health Sciences, Bursa Uludag University, Bursa, Turkey

**Keywords:** Aged, Cognitive dysfunction, Quality of Life, GERIATRIC MEDICINE, Exercise

## Abstract

**Objective:**

To compare the effects of cognitive-motor dual-task walking exercise and walking-only exercise on cognitive function and patient-centred outcomes in older adults with subjective brain fog.

**Design:**

Assessor-blinded, parallel-group randomised controlled trial.

**Setting:**

Outpatient clinics at a single university hospital in Istanbul, Türkiye.

**Participants:**

90 community-dwelling adults aged 65 years or older with subjective brain fog symptoms were randomly allocated to the exercise-only (EX; n=45) or cognitive-motor exercise (CM-EX; n=45) group. All participants completed the 4-week follow-up.

**Interventions:**

Both groups received supervised walking exercise three times weekly for 4 weeks. The CM-EX group performed cognitive tasks concurrently with walking.

**Primary and secondary outcome measures:**

The primary outcome was global cognitive function measured using the Montreal Cognitive Assessment (MoCA). Secondary outcomes were brain fog severity, depressive symptoms, sleep quality and quality of life, assessed using the Brain Fog Scale, Geriatric Depression Scale, Pittsburgh Sleep Quality Index and WHO Quality of Life Instrument-Older Adults Module (WHOQOL-OLD), respectively.

**Results:**

Both groups showed within-group improvements in MoCA scores (both p<0.001). The prespecified analysis of covariance (ANCOVA) estimated a baseline-adjusted between-group difference of −0.31 points (CM-EX minus EX 95% CI −1.38 to 0.76; p=0.566; partial eta squared (η²p)=0.004). An exploratory group-by-baseline MoCA interaction was observed (p=0.011), warranting cautious interpretation of the average adjusted difference. Brain fog severity decreased in both groups, with an exploratory baseline-adjusted between-group difference favouring EX (p=0.029; η²p=0.054). This finding should be interpreted cautiously because baseline scores differed between groups and no adjustment for multiple comparisons was applied. Depressive symptoms improved in both groups, with no significant between-group difference (p=0.069; η²p=0.038). Global sleep quality improved within EX (p=0.015) but not CM-EX (p=0.202); the between-group difference was not significant. WHOQOL-OLD total scores improved within EX (p=0.009), but not CM-EX (p=0.118), with no significant adjusted between-group difference (p=0.340; η²p=0.010).

**Conclusions:**

Cognitive-motor dual-task walking did not show superiority over walking-only exercise for global cognitive function or most secondary outcomes after 4 weeks. The exploratory MoCA interaction and short intervention period warrant cautious interpretation.

**Trial registration number:**

NCT07396779.

**STRENGTHS AND LIMITATIONS OF THIS STUDY**

- This study was designed as a prospectively registered, assessor-blinded randomised controlled trial with a parallel-group design, enhancing methodological transparency and reducing assessment bias.
- Both intervention protocols were supervised, standardised in frequency and duration and differed only in the addition of concurrent cognitive tasks, allowing a focused comparison between walking-only and cognitive-motor dual-task exercise.
- The study included multidimensional outcomes relevant to older adults with brain fog, including global cognitive function, brain fog severity, depressive symptoms, sleep quality and quality of life.
- The intervention period was limited to 4 weeks, and no long-term follow-up was conducted; therefore, the sustainability of the observed effects remains unclear.
- The absence of a non-exercise or usual-care control group limits attribution of the observed within-group changes to the exercise interventions.

## Background

 Brain fog is not regarded as a distinct diagnostic category, but rather as a cluster of subjective cognitive complaints commonly described as a ‘loss of mental clarity’. It is typically characterised by perceived difficulties in attention, information processing, memory and word-finding and is often accompanied by fatigue and mood-related symptoms. Because of heterogeneity in its definition and measurement, brain fog remains a complex transdiagnostic construct whose clinical significance cannot be fully captured by objective cognitive performance alone.^1^

In older adults, brain fog may present as cognitive slowing, distractibility, forgetfulness and mental cloudiness. Although such symptoms may overlap with early cognitive decline in some individuals, they should not be interpreted solely as indicators of neurodegenerative disease. Sleep disturbance, depressive and anxiety symptoms, chronic disease burden, medication use, fatigue and lifestyle-related factors may also contribute to this symptom experience. Therefore, brain fog in later life should be considered within a broader biopsychosocial framework rather than as a purely cognitive disorder.^2^

The clinical relevance of brain fog has increased in recent years, particularly in the context of post-infectious syndromes. In post-COVID-19 conditions, cognitive difficulties such as problems with thinking or concentration are frequently reported alongside fatigue, and global modelling studies have identified cognitive problems as one of the core long covid symptom clusters. Although brain fog is not specific to post-COVID-19, this literature has drawn attention to its potential impact on symptom burden, daily functioning and quality of life, especially among older adults with additional risk factors such as chronic disease burden, overweight or obesity.^3 4^

A key challenge is that subjective cognitive complaints do not consistently correspond to objective cognitive performance. Evidence suggests that subjective cognitive symptoms are often more strongly associated with fatigue, depression and anxiety than with cognitive test scores. This supports the need for interventions that address not only cognitive performance but also the fatigue, sleep and mood-related dimensions that may shape the experience of brain fog.^2^

Walking is increasingly recognised as a cognitively demanding activity in older adults, particularly under conditions requiring attention and executive control. In daily life, walking commonly occurs while individuals are simultaneously talking, monitoring the environment, shifting attention or performing mental tasks. Dual-task walking training, which combines motor and cognitive demands, may therefore provide an ecologically valid rehabilitation approach. Previous studies have shown that dual-task walking performance is sensitive to early cognitive decline and is associated with fall risk and functional limitations, while dual-task training may improve cognitive, physical and psychosocial outcomes in older adults.^5 6^

However, most existing studies have focused on cognitive impairment, fall risk or general physical function. The added value of cognitive-motor dual-task walking training over walking-only exercise remains unclear in older adults with brain fog, particularly when multidimensional outcomes such as cognitive function, brain fog severity, depressive symptoms, sleep quality and quality of life are considered together.^1 6 7^

Therefore, this study aims to compare the effects of cognitive-motor dual-task walking exercise and walking-only exercise on cognitive function, brain fog severity, depressive symptoms, sleep quality and quality of life in older adults with brain fog. We hypothesised that cognitive-motor dual-task walking training would produce greater improvements in global cognitive function, greater reductions in brain fog severity and more favourable effects on depressive symptoms, sleep quality and quality of life than walking-only exercise.

## Methods

### Study design and participants

This parallel-group, assessor-blinded randomised controlled trial was conducted at Istanbul Medipol University Hospital. Under the university’s policy, the committee’s remit includes exercise-related research on body physiology that does not require direct physician intervention. In this trial, a physician’s role was limited to eligibility screening, and a physiotherapist delivered the exercise interventions. The trial was registered at ClinicalTrials.gov (NCT07396779; initial release date: 1 February 2026). It was reported in accordance with the Consolidated Standards of Reporting Trials (CONSORT) statement, and the completed checklist is provided as online supplemental material.

Participants were recruited from outpatient clinics at the study hospital. Eligible participants were adults aged 65 years and older who reported subjective brain fog symptoms, including reduced attention, concentration or mental clarity, persisting for at least 4 weeks prior to enrolment. Brain fog was operationalised as a patient-reported, multidimensional symptom construct rather than a formal clinical diagnosis. Symptom severity was assessed using the Turkish Brain Fog Scale; no numerical cut-off was applied for eligibility. Additional inclusion criteria were a Montreal Cognitive Assessment (MoCA) score ≥20, the ability to follow instructions and participate in training, independent ambulation with or without an assistive device and no acute illness or medication changes within the preceding 4 weeks.

Eligibility screening aims to exclude individuals with diagnosed neurodegenerative disorders (eg, Alzheimer’s disease), a history of stroke or other major neurological conditions, acute confusional states (eg, delirium), severe psychiatric disorders or medical contraindications to exercise. Individuals with severe visual or hearing impairments that could interfere with assessment or training, as well as those unable to comply with study procedures, were also excluded.

Between 1 February and 22 April 2026, 156 individuals reporting brain fog symptoms were assessed for eligibility. Of these, 66 individuals were excluded before randomisation: 56 did not meet the inclusion criteria, 4 declined to participate and 6 were excluded for other reasons, including personal reasons (n=2), transportation difficulties (n=1) and language barriers (n=3). Therefore, 90 eligible participants were randomised to either the exercise-only group (EX; n=45) or the cognitive-motor exercise group (CM-EX; n=45). All 90 randomised participants completed the week-4 outcome assessment and were included in the final analyses (figure 1).

**Figure 1:**
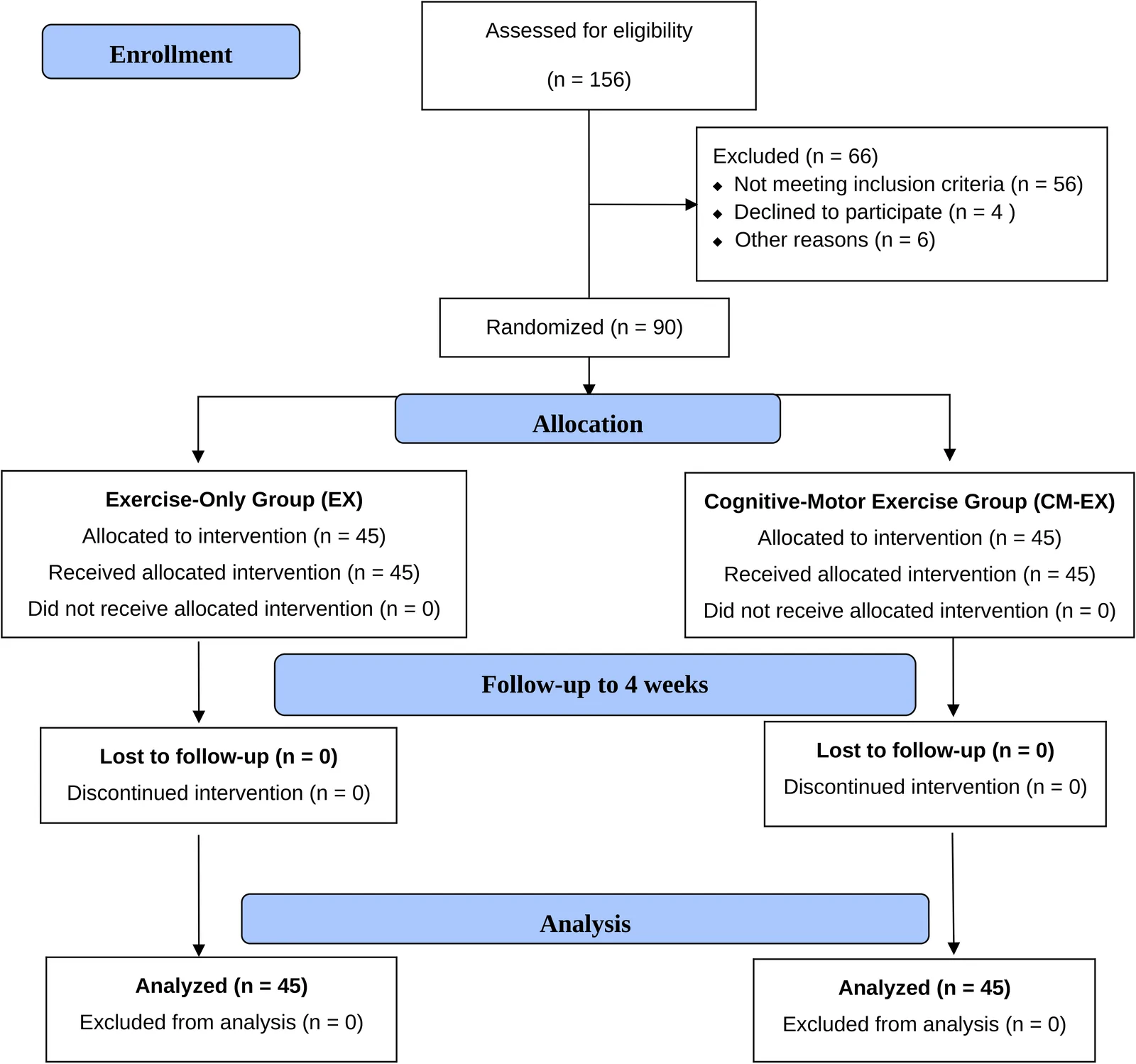
Consolidated Standards of Reporting Trials (CONSORT) participant flow diagram.

### Randomisation and blinding

Participants who met the eligibility criteria and provided written informed consent were randomly allocated in a 1:1 ratio to the EX or CM-EX group. An independent researcher who was not involved in recruitment, intervention delivery or outcome assessment generated the allocation sequence before enrolment using Research Randomizer (https://www.randomizer.org/). The same researcher prepared sequentially numbered, opaque, sealed envelopes to conceal allocation. No stratification variables were used. After each participant’s baseline assessment was completed, the treating physiotherapist opened the next envelope in sequence and delivered the allocated intervention.

Participants and the treating physiotherapist were not blinded because of the nature of the interventions. All baseline and post-intervention assessments were performed by an outcome assessor who did not deliver the interventions and remained blinded to group allocation. Participants were instructed not to disclose their intervention to the assessor. An independent statistician analysed data coded as groups A and B, without access to intervention descriptions or the correspondence between group codes and interventions until the primary analyses were completed. The treating physiotherapist retained this information.

### Interventions

The interventions were delivered individually to both groups under physiotherapist supervision, three times per week for 4 weeks. Exercises were prescribed at a low intensity and individualised according to each participant’s physical capacity, chronic disease history, ambulatory status and balance performance. Participant safety, exercise tolerance, balance status and fatigue were monitored throughout all sessions. Because individual exercise capacity and physiological responses to exercise may vary considerably among older adults, exercise intensity was individually determined and adjusted according to each participant’s performance and exercise tolerance. Exercise intensity was guided by the Borg Rating of Perceived Exertion scale (6–20), with a target range of 9–11, corresponding to light exertion and consistent with the low-intensity exercise prescription. Participants were regularly asked to rate their perceived exertion during the exercise sessions, and the physiotherapist used these responses together with observed fatigue, gait and balance performance and overall exercise tolerance to determine whether the exercise intensity should be maintained or adjusted. The exercise intensity was reduced, the pace was slowed or a brief rest period was provided when participants reported excessive exertion or demonstrated signs of fatigue or reduced exercise tolerance. Thus, although the exercise protocol and duration were standardised across participants, the intensity and pace of individual exercises were adapted to each participant’s functional capacity while avoiding excessive fatigue and maintaining safe participation. Brief rest periods, assistive devices, environmental support and minor adjustments to pace or task difficulty were permitted when clinically required to ensure safe and feasible participation.

Each session consisted of a 5-min warm-up, a 25-min main walking phase and a 5-min cool-down. The warm-up and cool-down included marching in place and slow-paced straight-line walking. Session frequency, total duration, supervision, exercise sequence and the core walking structure were standardised across groups. The only between-group difference was the addition of concurrent cognitive tasks during walking in the CM-EX group. Missed sessions were rescheduled to support completion of the prescribed 12-session programme.

### EX group

Participants in the EX group performed low-intensity walking-based motor exercises without any concurrent cognitive task. The intervention was designed to support safe mobility, improve postural control and enhance functional walking performance in older adults. The 25-min main walking phase consisted of five exercises, each performed for approximately 5 min: tandem walking, side walking, figure-of-eight walking, backward walking and walking while carrying a light object.

Tandem walking was used to challenge dynamic postural stability and mediolateral balance control. Side walking targeted lateral stability, weight transfer and lower-extremity coordination. Figure-of-eight walking was included to challenge directional changes, turning control and spatial gait regulation. Backward walking addressed step control, cautious weight shifting and balance strategies. In the walking-while-carrying-an-object task, participants walked along a straight path while carrying a light object, such as a plastic cup containing water or a small ball, to simulate a functionally relevant daily-life motor demand. All tasks were performed in a safe environment, and assistive devices or external support were permitted when necessary. No structured concurrent cognitive task was added to the walking exercises.

### CM-EX group

Participants in the CM-EX group performed the same walking-based motor protocol as the EX group in terms of session frequency, total duration and motor content; however, concurrent cognitive tasks were incorporated throughout the main walking phase. This dual-task approach was designed to simultaneously target motor performance and cognitive domains commonly affected in older adults with brain fog, including attention, working memory, verbal fluency, sequencing and executive function.

During the main phase, the same five walking exercises were performed but each was paired with a simultaneous cognitive demand. Cognitive tasks were delivered verbally by the supervising physiotherapist and selected from a standardised task pool. The cognitive task pool and the type of cognitive demand paired with each walking exercise were standardised across participants. However, cognitive-task performance was not formally scored or recorded using a separate cognitive performance measure during the intervention. Instead, task difficulty and progression were determined clinically by the supervising physiotherapist based on the participant’s ability to perform the cognitive task while maintaining safe and adequate walking performance. When a participant demonstrated adequate performance and could simultaneously maintain gait and postural stability, the cognitive task was progressed by increasing task complexity or cognitive demand. Conversely, when a participant demonstrated difficulty completing the cognitive task, marked slowing of gait, loss of balance or excessive cognitive or physical fatigue, the task was simplified or temporarily paused. Thus, progression of cognitive-task difficulty was individualised according to each participant’s performance and safety rather than being based on a predefined numerical progression criterion. During tandem walking, participants performed forward counting, backward counting from a given number or continuation of sequential number series. During side walking, verbal fluency tasks were used, such as generating words beginning with a specific letter or naming items within a semantic category. During figure-of-eight walking, participants recited the days of the week or months of the year in forward or reverse order. During backward walking, backward counting or other simple mentally demanding tasks were administered, with task complexity reduced when necessary to preserve gait safety. During the walking-while-carrying-an-object task, participants performed concurrent verbal tasks such as category counting, simple recall or sequencing activities to simulate functionally relevant dual-task demands commonly encountered in daily life.

Throughout the intervention, priority was consistently given to gait safety and postural stability. When participants demonstrated marked difficulty, the cognitive task was simplified, briefly paused or followed by a short rest period, while the overall structure of the intervention was maintained.

### Outcome measures

Outcome assessments were performed for all participants at baseline and at the end of the 4-week intervention period by an assessor blinded to group allocation.

### Primary outcome

#### Cognitive function

Cognitive function was assessed using the MoCA, a widely used screening tool for detecting mild cognitive impairment. The MoCA evaluates multiple cognitive domains, including executive function, visuospatial ability, memory, attention, language, abstraction, delayed recall and orientation. The total score ranges from 0 to 30, with higher scores indicating better cognitive performance. The MoCA has demonstrated high sensitivity and diagnostic accuracy for identifying mild cognitive impairment and early cognitive decline in older adults and is widely used in both clinical and research settings.^8 9^

### Secondary outcomes

#### Brain fog severity

Brain fog severity was assessed using the Brain Fog Scale, developed by Atik and İnel Manav. This self-reported scale consists of 30 items across three subdomains (cognitive, physical and psychological symptoms), rated on a 5-point Likert scale. Higher scores indicate greater brain fog severity. The scale has demonstrated satisfactory psychometric properties and is suitable for evaluating multidimensional subjective cognitive complaints in clinical populations.^10^

#### Depressive symptoms

Depressive symptoms were evaluated using the Geriatric Depression Scale (GDS), a validated self-report instrument specifically developed for older adults. The scale consists of 30 dichotomous (yes/no) items and minimises the influence of somatic symptoms. Total scores range from 0 to 30, with higher scores indicating greater depressive symptom severity. The GDS has been widely validated and remains one of the most commonly used screening tools for depression in geriatric populations.^11 12^

#### Sleep quality

Sleep quality was assessed using the Pittsburgh Sleep Quality Index (PSQI), which evaluates subjective sleep quality and disturbances over the past month. The PSQI includes 19 items grouped into seven components, each scored from 0 to 3. The global score ranges from 0 to 21, with higher scores indicating poorer sleep quality.^13 14^

#### Quality of life

Quality of life was evaluated using the WHO Quality of Life Instrument-Older Adults Module (WHOQOL-OLD). This instrument consists of 24 items across six domains: sensory abilities, autonomy, past-present-future activities, social participation, death and dying and intimacy. Each item is rated on a Likert scale, and higher scores indicate better perceived quality of life. The WHOQOL-OLD has demonstrated good psychometric properties and cross-cultural validity in older populations.^15 16^

### Statistical analysis

An a priori power analysis was conducted using G*Power (V.3.1) for the primary outcome, global cognitive function assessed by the MoCA. The analysis was based on an analysis of covariance (ANCOVA) model for a baseline-adjusted between-group comparison, with two groups and one covariate (baseline MoCA score). A standardised effect size (ES) of f=0.30 was assumed a priori for the primary comparison. This value lies between Cohen’s conventional medium (f=0.25) and large (f=0.40) effect-size benchmarks.^17^ With an α level of 0.05 and 80% power (1-β=0.80), the required total sample size was estimated as 90 participants. Although the planned recruitment target was increased to 100 participants to account for possible attrition or incomplete data, 90 participants were ultimately randomised, and all completed the study. This final sample therefore met the minimum sample size requirement defined by the a priori power analysis.

All statistical analyses were performed using IBM SPSS Statistics (V.22.0; IBM, Armonk, New York, USA). The normality of continuous variables was assessed using the Shapiro-Wilk test. Based on the results of the normality assessments, parametric tests were applied for outcomes that met the assumption of normality, whereas non-parametric methods were used for variables that violated this assumption. Descriptive statistics are presented as mean±SD or median (IQR) for continuous variables, depending on distributional characteristics, and as frequency (percentage) for categorical variables. Baseline demographic and clinical characteristics were compared between groups using the Mann-Whitney U test for continuous variables and the χ^2^ test or Fisher’s exact test, as appropriate, for categorical variables. For outcomes that satisfied the normality assumption or showed acceptable distributional characteristics for parametric analysis, including MoCA, Brain Fog Scale, GDS and WHOQOL-OLD scores, within-group changes were analysed using paired-samples t-tests, and unadjusted between-group comparisons at baseline and post-intervention were performed using independent-samples t-tests. Baseline-adjusted between-group comparisons at week 4 were conducted using ANCOVA, with the corresponding baseline score included as a covariate. Model assumptions, including linearity, homogeneity of regression slopes, residual normality and homoscedasticity, were assessed. For the primary MoCA outcome, homogeneity of regression slopes was specifically assessed using a group-by-baseline MoCA interaction term. Because this interaction was statistically significant, indicating violation of the parallel-slopes assumption, the prespecified ANCOVA estimate was interpreted cautiously. A model including the group-by-baseline MoCA interaction was additionally fitted as an exploratory sensitivity analysis to examine whether the between-group effect varied according to baseline cognitive performance. ANCOVA ESs were reported as partial eta squared (η²p).

Sleep-related outcomes assessed with the PSQI were analysed using non-parametric methods. Within-group changes were examined using the Wilcoxon signed-rank test, and between-group comparisons were performed using the Mann-Whitney U test. ESs for within-group changes were calculated as r=Z/√N and interpreted accordingly. For interpretation, η²p values of 0.01, 0.06 and 0.14 and r values of 0.10, 0.30 and 0.50 were considered small, medium and large, respectively.^18 19^ Global cognitive function assessed by the MoCA was defined as the primary outcome. All other outcomes were secondary and their findings were interpreted as exploratory. Because multiple secondary comparisons were performed, no formal multiplicity adjustment was applied; therefore, these findings should be interpreted as exploratory rather than confirmatory.

Literature-based interpretability thresholds were used to contextualise changes in MoCA (approximately 1–2 points) and PSQI (approximately 3 points), rather than as formal statistical decision thresholds.^14 20 21^ Changes in WHOQOL-OLD scores were interpreted descriptively, without applying a clinical importance threshold. All 90 randomised participants completed the intervention according to the protocol and were analysed in their assigned groups. The intention-to-treat and per-protocol populations therefore comprised the same participants. Outcome data were complete at baseline and week 4; no imputation was required.

### Patient and public involvement

Patients and/or the public were not involved in the design, conduct, reporting or dissemination plans of this research.

## Results

Baseline demographic and clinical characteristics are presented in table 1. Baseline Brain Fog Scale scores were higher in the CM-EX group than in the EX group (table 2).

**Table 1:** Baseline demographic and clinical characteristics of participants across intervention groups

Variables	EX group (n=45) median (IQR)	CM-EX group (n=45) median (IQR)	P value
Age (years)	68 (67 to 70)	68 (65 to 72)	0.370*
Height (cm)	165 (160 to 175)	164 (159 to 172)	0.297*
Weight (kg)	75 (68 to 81)	78 (68 to 90)	0.185*
BMI	25.4 (24.6 to 29.7)	27.9 (24.7 to 33.05)	0.125*
Gender (n, %)			0.671†
Female	24 (53.3%)	27 (60.0%)	
Male	21 (46.7%)	18 (40.0%)	
Presence of chronic disease (n, %)			1.000†
Yes	27 (60.0%)	27 (60.0%)	
No	18 (40.0%)	18 (40.0%)	
Smoking status (n, %)			0.352†
Yes	4 (8.9%)	8 (17.8%)	
No	41 (91.1%)	37 (82.2%)	
Alcohol consumption in the past 4 weeks, n (%)			1.000‡
Yes	4 (8.9%)	3 (6.7%)	
No	41 (91.1%)	42 (93.3%)	
Number of medications used	2 (1 to 3)	3 (2 to 5)	0.055*
Number of days of regular exercise per week	0 (0 to 0)	0 (0 to 0)	1.000*

P<0.05 was considered statistically significant.

* Mann-Whitney U test.

† χ2 test.

‡ Fisher’s exact test.

BMI, Body Mass Index; CM-EX group, cognitive-motor exercise group (walking exercises combined with concurrent cognitive tasks); EX group, exercise-only group (walking exercises without concurrent cognitive tasks).

**Table 2:** Within-group and between-group analyses of cognitive and psychosocial outcome measures before and after the intervention

Variables	Group	Baseline (mean±SD)	4 week (mean±SD)	p*	p†	η²p
Total MoCA score	EX group	24.38±2.41	26.49±2.78	<0.001	0.566	0.004
	CM-EX group	25.27±2.90	26.60±2.84	<0.001		
	p‡	0.118	0.852			
Total brain fog score	EX group	66.82±11.50	55.98±14.37	<0.001	0.029	0.054
	CM-EX group	75.02±22.52	68.22±21.79	0.003		
	p‡	0.032	0.002			
Total GDS score	EX group	10.96±5.08	8.02±4.39	<0.001	0.069	0.038
	CM-EX group	10.98±5.78	9.07±4.90	<0.001		
	p‡	0.985	0.289			

ESs are reported as η²p for ANCOVA.

P<0.05 was considered statistically significant.

* Paired sample t-test for within-group comparisons.

† ANCOVA for baseline-adjusted post-intervention between-group comparisons.

‡ Independent samples t-test for unadjusted between-group comparisons at baseline and post-intervention.

ANCOVA, analysis of covariance; CM-EX group, cognitive-motor exercise group (walking exercises combined with concurrent cognitive tasks); ES, effect size; EX group, exercise-only group (walking exercises without concurrent cognitive tasks); GDS, Geriatric Depression Scale; MoCA, Montreal Cognitive Assessment; η²p, partial eta squared.

Cognitive and psychosocial outcomes are summarised in table 2. Both groups demonstrated significant within-group improvements in MoCA following the 4-week intervention (both p<0.001). The prespecified ANCOVA showed no significant baseline-adjusted between-group difference (CM-EX minus EX: −0.31 points; 95% CI −1.38 to 0.76; p=0.566; η²p=0.004). In the exploratory interaction model, the group-by-baseline MoCA interaction was statistically significant (β=0.514; 95% CI 0.120 to 0.908; p=0.011), indicating that the parallel-slopes assumption was not met and that the between-group difference may vary according to baseline cognitive performance. Therefore, the prespecified ANCOVA estimate should be interpreted cautiously, and no definitive between-group superiority can be concluded from this analysis. Brain fog scores decreased significantly in both groups (EX: p<0.001; CM-EX: p=0.003). Baseline brain fog severity was significantly higher in the CM-EX group than in the EX group. The exploratory baseline-adjusted ANCOVA indicated a between-group difference favouring EX (p=0.029; η²p=0.054). Given the baseline imbalance in brain fog severity and the absence of adjustment for multiple comparisons, this secondary outcome finding should be interpreted as exploratory rather than confirmatory. GDS scores improved significantly within both groups (both p<0.001), with no significant between-group difference after baseline adjustment (p=0.069; η²p=0.038).

Sleep-related outcomes (PSQI) are presented in table 3. Within-group improvements were observed in several PSQI components in the EX group, including sleep latency, sleep disturbances, daytime dysfunction and the global PSQI score, whereas the CM-EX group showed a significant improvement only in subjective sleep quality. However, no significant between-group differences were observed for PSQI components or the global PSQI score at baseline or post-intervention (all p>0.05). Quality-of-life outcomes (WHOQOL-OLD) are presented in table 4. The EX group showed significant within-group improvements in several domains and in the total score, whereas no significant within-group changes were observed in the CM-EX group. Baseline differences were present in the WHOQOL-OLD total score and in the domains of past, present and future activities, social participation and death and dying; however, baseline-adjusted ANCOVA analyses showed no significant between-group differences at week 4, across the domains or total score (all p>0.05). No adverse events or unintended effects were reported in either group during the 4-week intervention period.

**Table 3:** Within-group and between-group analyses of sleep-related outcome measures before and after the intervention

Variables	Group	Baseline median (IQR)	4 week median (IQR)	p*	r
PSQI					
Subjective sleep quality	EX group	1 (1 to 1)	1 (1 to 1)	0.060	0.19
	CM-EX group	1 (1 to 2)	1 (0 to 1)	0.005	0.55
	p†	0.859	0.784		
Sleep latency	EX group	2 (1 to 2)	1 (1 to 2)	0.002	0.52
	CM-EX group	1 (1 to 2)	1 (0 to 2)	0.660	0.12
	p†	0.076	0.869		
Sleep duration	EX group	0 (0 to 1)	0 (0 to 1)	0.825	0.06
	CM-EX group	0 (0 to 1)	0 (0 to 1)	0.869	0.05
	p†	0.824	0.945		
Habitual sleep efficiency	EX group	0 (0 to 0)	0 (0 to 1)	1.000	0.00
	CM-EX group	0 (0 to 1)	0 (0 to 1)	0.439	0.20
	p†	0.065	0.258		
Sleep disturbances	EX group	2 (1 to 2)	1 (1 to 2)	<0.001	0.71
	CM-EX group	2 (1 to 2)	2 (1 to 2)	0.599	0.09
	p†	0.727	0.077		
Use of sleeping medication	EX group	0 (0 to 0)	0 (0 to 0)	0.391	0.15
	CM-EX group	0 (0 to 0)	0 (0 to 0)	0.655	0.08
	p†	0.122	0.055		
Daytime dysfunction	EX group	0 (0 to 1)	0 (0 to 1)	0.007	0.49
	CM-EX group	1 (0 to 1)	0 (0 to 1)	0.399	0.18
	p†	0.644	0.129		
Global PSQI score	EX group	6 (5 to 8)	5 (4 to 8)	0.015	0.46
	CM-EX group	6 (4 to 10)	5 (3 to 9)	0.202	0.22
	p†	0.548	0.919		

r: Effect size for within-group comparisons using the Wilcoxon signed-rank test, calculated as Z/√N, where Z is the standardized test statistic and N is the number of observations used in the calculation.

P<0.05 was considered statistically significant.

* Wilcoxon signed-rank test for within-group comparisons.

† Mann-Whitney U test for between-group comparisons at baseline and post-intervention.

CM-EX group, cognitive-motor exercise group (walking exercises combined with concurrent cognitive tasks); ES, effect size; EX group, exercise-only group (walking exercises without concurrent cognitive tasks); PSQI, Pittsburgh Sleep Quality Index.

**Table 4:** Within-group and between-group analyses of quality-of-life outcome measures before and after the intervention

Variables	Group	Baseline (mean±SD)	4 week (mean±SD)	p*	p†	η²p
WHOQOL-OLD						
Sensory abilities	EX group	14.00±3.04	15.13±2.07	0.021	0.103	0.030
	CM-EX group	14.64±2.78	14.89±2.85	0.512		
	p‡	0.297	0.643			
Autonomy	EX group	13.93±3.05	14.58±3.19	0.084	0.501	0.005
	CM-EX group	14.76±2.83	14.96±2.80	0.343		
	p‡	0.188	0.552			
Past, present and future activities	EX group	14.18±2.64	15.07±2.23	0.013	0.779	0.001
	CM-EX group	16.18±2.25	16.38±2.34	0.281		
	p‡	<0.001	0.008			
Social participation	EX group	12.67±3.10	14.00±2.88	0.006	0.272	0.014
	CM-EX group	14.33±2.76	14.62±2.53	0.198		
	p‡	0.008	0.280			
Death and dying	EX group	13.91±1.86	14.87±2.19	0.041	0.212	0.018
	CM-EX group	14.89±2.26	15.02±2.12	0.304		
	p‡	0.027	0.733			
Intimacy	EX group	14.40±2.43	14.58±1.90	0.771	0.877	<0.001
	CM-EX group	14.36±2.41	14.60±2.49	0.882		
	p‡	0.931	0.962			
Total score	EX group	83.09±13.97	88.22±12.21	0.009	0.340	0.010
	CM-EX group	89.16±11.82	90.47±11.40	0.118		
	p‡	0.029	0.369			

ESs are reported as η²p for ANCOVA.

P<0.05 was considered statistically significant.

* Paired sample t-test for within-group comparisons.

† ANCOVA for baseline-adjusted post-intervention between-group comparisons.

‡ Independent-samples t-test for unadjusted between-group comparisons at baseline and post-intervention.

ANCOVA, analysis of covariance; CM-EX group, cognitive-motor exercise group (walking exercises combined with concurrent cognitive tasks); ES, effect size; EX group, exercise-only group (walking exercises without concurrent cognitive tasks); WHOQOL-OLD, WHO Quality of Life Instrument-Older Adults Module; η²p, partial eta squared.

Clinical relevance was examined using published interpretability thresholds where available. For MoCA, anchor-based evidence suggests that a 1–2-point improvement may be clinically meaningful, and the within-group gains in both groups were within or above this range.^20 21^ For PSQI, a reduction of approximately 3 points is often considered clinically meaningful; the median change in global PSQI did not clearly reach the commonly cited 3-point threshold.^14^ WHOQOL-OLD score changes were interpreted descriptively and were not classified as clinically meaningful on the basis of a numerical threshold. No population-specific minimal clinically important difference was applied to Brain Fog Scale or GDS scores in this study.

## Discussion

In this randomised controlled trial, we compared the effects of cognitive-motor dual-task walking training with walking-only exercise on cognitive and patient-centred outcomes in community-dwelling adults aged 65 years and older who had subjective brain fog symptoms. Both interventions were associated with improvements in global cognitive function and depressive symptoms, with no significant between-group differences. Similarly, no significant group differences were observed for sleep quality or quality of life. The exploratory baseline-adjusted comparison of brain fog severity favoured EX, but this secondary finding should be interpreted cautiously because of baseline imbalance and the absence of adjustment for multiple comparisons. Without a non-exercise control group, the observed within-group changes cannot be attributed specifically to the shared exercise components.

With ageing, walking may become increasingly dependent on executive resources and attentional control, particularly under dual-task conditions, which are associated with greater prefrontal cortical activation.^22^

Therefore, dual-task walking training may theoretically enhance divided attention and executive control by simulating real-life cognitive-motor demands. However, such benefits may depend on the degree of cognitive load progression, task prioritisation strategies and whether the total intervention dose is sufficient to induce measurable neuroplastic adaptation. Previous evidence suggests that dual-task training may produce small-to-moderate improvements in global cognition, executive function and gait, particularly in populations with more evident cognitive impairment.^23^

Regarding global cognitive function, both groups demonstrated significant improvements in MoCA scores, but no statistically significant average between-group difference was detected. In the Synchronizing Exercises, Remedies in Gait and Cognition randomised trial, adding sequential computerised cognitive training to aerobic-resistance exercise produced greater improvements in Alzheimer's Disease Assessment Scale–Cognitive Subscale scores than exercise alone in older adults with mild cognitive impairment.^24^ However, differences in participant characteristics, intervention duration and training format limit direct comparison with the present 4-week simultaneous cognitive-motor walking intervention. Moreover, cognitive gains from walking programmes may be more pronounced in protocols lasting at least 6–26 weeks and delivered three or more times per week for longer session durations.^25^ Therefore, the absence of a between-group difference in the present study may be related to the relatively short intervention period, the active nature of both interventions and the use of MoCA as a global screening tool rather than a domain-specific neuropsychological assessment. However, repeated administration of the same MoCA within a 4-week interval may have introduced a practice effect, which could have contributed to the observed within-group improvements.

Although some studies have reported superior cognitive effects of dual-task training, these benefits are often observed in older adults with more pronounced cognitive impairment or in longer and more intensive protocols.^1 6 23^ This distinction is important because the present sample consisted of individuals with subjective brain fog and relatively preserved global cognition. The additional cognitive benefit of dual-task training may be more detectable in populations with lower cognitive reserve or more evident cognitive impairment.^23^

For total brain fog severity, both groups showed within-group improvements, and the exploratory baseline-adjusted comparison favoured walking-only exercise. However, brain fog severity was a secondary outcome, baseline scores were higher in the CM-EX group, and no adjustment for multiple comparisons was applied; therefore, this finding should be considered hypothesis-generating rather than confirmatory evidence of superiority. One possible explanation is that walking-only exercise may have imposed a lower perceived cognitive burden than dual-task training, which could be relevant in individuals whose primary complaint is subjective mental fatigue or reduced mental clarity. Brain fog is a multidimensional subjective experience involving attention, mental clarity, fatigue and affective burden; therefore, changes in brain fog severity may not necessarily parallel changes in objective cognitive performance. Existing evidence also indicates that subjective cognitive complaints are only weakly associated with objective cognitive measures and may be more strongly influenced by psychological distress, fatigue and symptom perception.^1 2^ Thus, the additional cognitive demands imposed by dual-task training may have limited the reduction in perceived symptom burden during the short intervention period. Since benefits of dual-task training may require longer and more progressive protocols, particularly in individuals with more evident cognitive impairment, this result should not be interpreted as evidence against cognitive-motor training.^6 23^ Moreover, because no established minimal clinically important difference is available for the Brain Fog Scale, the clinical relevance of this between-group difference should be interpreted cautiously.^2 23^

Depressive symptoms improved significantly in both groups, without a between-group difference. The within-group reductions in depressive symptoms may reflect shared intervention components or non-specific effects; however, their causes cannot be established without a non-exercise control group. This finding is consistent with evidence showing that exercise-based interventions can reduce depressive symptoms in older adults.^26 27^ However, our hypothesis that dual-task training would provide additional benefits for depressive symptoms was not supported. One possible explanation is the short intervention duration, as depression is a complex biopsychosocial outcome and may require longer and progressively structured interventions to demonstrate differential effects. Exercise may influence depression through reduced inflammation, neurotransmitter regulation, stress modulation and enhanced neuroplasticity, but these mechanisms generally require sufficient intervention duration and dose to translate into clinically meaningful between-group differences.^28 29^ Median global PSQI scores decreased in both groups, but the within-group change was statistically significant only in EX. No significant between-group difference was detected. Previous systematic reviews and meta-analytic evidence indicate that exercise interventions can improve subjective sleep quality in older adults, including when measured using the PSQI.^30^

The absence of a detectable additional dual-task benefit may be explained by the short duration of the protocol, the relatively broad and multifactorial nature of sleep quality and the possibility that a higher or longer cognitive-motor training dose is required to influence sleep-related outcomes. A systematic review and meta-analysis suggested that Qigong may improve subjective sleep quality in older adults, although the certainty of evidence for the PSQI total score was low and heterogeneity was substantial.^31^ Baseline-adjusted analyses showed no significant between-group differences in quality-of-life outcomes at week 4. This may reflect the fact that both interventions shared therapeutic components likely to influence quality of life, including regular physical activity, supervised participation and support for functional mobility. Previous studies indicate that exercise interventions can improve quality of life in older adults, but differences between active exercise modalities are not always evident.^32^ Although combined physical-cognitive or dual-task approaches may improve quality of life in higher-risk groups such as those with cognitive frailty or mild cognitive impairment, these effects are more commonly reported in longer and more intensive interventions.^33^ In community-dwelling older adults with subjective brain fog and relatively preserved function, a 4-week intervention may be insufficient to produce clear between-group differences in such a broad multidimensional outcome.^34 35^ From a clinical perspective, walking-only exercise may represent a potential exercise option for older adults with subjective brain fog, while cognitive-motor dual-task training may require longer duration, progressive task difficulty and more targeted participant selection to demonstrate additional benefits. Its role relative to usual care, as well as its feasibility and cost-effectiveness, requires further evaluation.

This study has several limitations. First, recruitment from a single hospital’s outpatient clinics and eligibility criteria requiring preserved cognitive and ambulatory capacity and clinical stability may limit generalisability to the broader population of older adults with subjective brain fog. An exploratory group-by-baseline MoCA interaction suggested that the ANCOVA parallel-slopes assumption was not met; therefore, the prespecified average adjusted estimate should be interpreted cautiously. Future trials should assess whether this interaction is reproducible before conclusions are drawn about baseline-dependent treatment effects. Second, the intervention lasted only 4 weeks, and no long-term follow-up was conducted; therefore, the sustainability of the observed effects remains unknown. In addition, the study did not include a non-exercise or usual-care control group. Therefore, the observed changes cannot be attributed exclusively to the exercise interventions, as potential effects of time, repeated assessment, attention or other non-specific factors cannot be completely excluded. Third, although global cognition was assessed using the MoCA, detailed neuropsychological tests targeting attention, executive function, processing speed and working memory were not included. As a result, domain-specific cognitive changes may not have been fully captured. Finally, no adjustment for multiple comparisons was applied to the secondary outcomes; therefore, these findings, including the between-group difference in brain fog severity, should be interpreted as exploratory rather than confirmatory. Future multicentre trials with longer, progressive intervention protocols, domain-specific cognitive assessments and follow-up evaluations are needed to clarify the added value of cognitive-motor dual-task training in older adults with brain fog.

Another limitation is that most of the secondary outcomes were assessed using self-reported measures. Although these instruments are widely used and provide valuable information regarding participants’ perceived symptoms and functional status, self-reported outcomes may be influenced by participants’ expectations, perceptions of treatment benefit and response tendencies. Therefore, the possibility of subjective reporting or expectation-related effects cannot be completely excluded. Future studies should consider combining self-reported measures with more objective behavioural, cognitive or physiological measures to provide a more comprehensive assessment of intervention effects.

## Conclusions

This randomised controlled trial suggests that both walking-only exercise and cognitive-motor dual-task walking exercise were associated with improvements in global cognitive function in older adults with subjective brain fog. However, cognitive-motor dual-task walking training did not demonstrate superior effects over walking-only exercise after 4 weeks. Given the absence of a non-exercise control group, these improvements cannot be attributed specifically to the exercise interventions. Structured, supervised walking-based exercise may warrant further investigation in this population. Longer, progressively structured trials with follow-up assessments are needed to clarify whether dual-task training provides additional benefits beyond walking exercise alone.

## Supplementary material

10.1136/bmjopen-2026-121892 online supplemental file 1

10.1136/bmjopen-2026-121892 online supplemental file 2

## Data Availability

Data are available upon reasonable request.
