# Supplementary material for: Effects of cognitive-motor dual-task walking exercise versus walking-only exercise on cognitive function and patient-centred outcomes in older adults with subjective brain fog: a randomised controlled trial
Source: BMJ Open. 2026 Oct 20;16(10):e121892. doi: 10.1136/bmjopen-2026-121892 (PMC13684161; doi:10.1136/bmjopen-2026-121892)
Supplement: Supplementary file 1 [file bmjopen-2026-121892.reviewer_comments.pdf]

PEER REVIEW HISTORY

BMJ Open publishes all reviews undertaken for accepted manuscripts. Reviewers are asked to complete a checklist review form and are provided with free text boxes to elaborate on their assessment. These free text comments are reproduced below.

ARTICLE DETAILS

Title (Provisional)

Effects of Cognitive-Motor Dual-Task Walking Exercise Versus Walking-Only Exercise on Cognitive Function and Patient-Centered Outcomes in Older Adults with Subjective Brain Fog: A Randomized Controlled Trial

Authors

DANSUK, EMRE; MENEK, BURAK

VERSION 1 - REVIEW

**Reviewer**	**1**
**Name**	**Dongaz, Ozge Ipek**
**Affiliation**	**Mugla Sitki Kocman Universitesi**
**Date**	**20-Jul-2026**
**COI**	**None**

Dear Authors,

Thank you for your effort. This is a well-written, clinically relevant, and methodologically sound randomized controlled trial. The comparison of walking-only and cognitive–motor exercise provides valuable information for the rehabilitation of older adults reporting brain fog symptoms. I have only a few comments that may improve the clarity and reproducibility of the study:

Please explain how the exercise intensity was determined, individualised, and monitored during the intervention.

Please clarify how the difficulty of the cognitive tasks was standardised and progressed across participants. Was cognitive-task performance formally recorded and used to guide progression, or were adjustments based solely on the physiotherapist’s clinical judgement?

Please provide further information regarding intervention adherence. Did all participants complete every planned exercise session? Reporting the number or proportion of sessions completed, missed sessions, and any modifications or interruptions would improve the transparency of the intervention.

Most secondary outcomes were based on self-reported measures. Please acknowledge in the limitations that these outcomes may have been influenced by participants' expectations and subjective reporting.

Overall, this is a valuable and clearly presented study. I believe that addressing these minor points would further strengthen the manuscript.

With warm wishes,

**Reviewer**	**2**
**Name**	**Polat, Hakan**
**Affiliation**	**Sanko University, Department of Physiotherapy and Rehabilitation**
**Date**	**24-Jul-2026**
**COI**	**None**

Dear Author,

This manuscript addresses a timely and clinically relevant topic by comparing cognitive–motor dual-task walking with a matched walking-only intervention in older adults experiencing subjective brain fog. The direct comparison of two active exercise programmes, together with the inclusion of cognitive, psychological, sleep-related and quality-of-life outcomes, represents a valuable contribution to the current literature. The assessor-blinded randomized design, prospective trial registration and standardized intervention duration are additional strengths.

The findings are also clinically informative, particularly because the addition of cognitive tasks did not produce superior short-term effects compared with walking-only exercise. This negative comparative finding is relevant and may help guide the design of future dual-task interventions. The manuscript is generally well written and logically structured. I recommend publication after the following minor revisions.

Lines 26–27: The title should define the study population more precisely, as participants were specifically older adults with subjective brain fog. Please also consider replacing "cognitive task-based" with the more established term "cognitive–motor dual-task."

Lines 50–52: Brain fog severity is a secondary outcome, baseline scores differed between groups, and no adjustment for multiple comparisons was applied. Please clearly describe this finding as exploratory rather than confirmatory.

Lines 69–80: This section acknowledges the short intervention period and the lack of follow-up assessment. However, the absence of a non-exercise or usual-care control group should also be reported as an important limitation.

Lines 140–148: The manuscript states that participants were required to have moderate or greater symptom severity according to the Turkish Brain Fog Scale; however, the exact cut-off score is not reported. Please specify the operational cut-off and its supporting reference. If no validated cut-off exists, revise the wording and clarify that the scale was used to quantify symptom severity rather than classify participants into severity categories.

Lines 157–164: It is unclear whether the withdrawals occurred before or after randomization. Please clarify the participant flow, especially since all randomized participants completed follow-up.

Lines 184–193: The exercises are described as low intensity; however, it is unclear how exercise intensity was determined or monitored. Please clarify the method used.

Lines 326–327: The absence of statistically significant baseline differences does not establish group comparability. Please revise this statement and note that baseline Brain Fog Scale scores differed between groups.

Lines 349–358: Although the 1–2 point increase in MoCA scores may be clinically meaningful, repeated administration of the same test within four weeks may have introduced a practice effect. Please acknowledge this possibility in the discussion.

Lines 483–486: Referring to walking-only exercise as a “pragmatic first-line strategy” may overstate the findings, as usual care, feasibility, and cost-effectiveness were not assessed. Please use more cautious wording.

Lines 499–506: The terms “well-tolerated” and “low-cost” are not sufficiently supported by adherence, adverse-event, or cost data. Please use more cautious wording and avoid causal conclusions in the absence of a non-exercise control group.

Lines 514–516: The data availability statement does not specify which data will be shared, the availability period, or the conditions for access. Please provide these details and ensure consistency with the trial registration.

**Reviewer**	**3**
**Name**	**Kelson, Zoe**
**Affiliation**	**University of Exeter, Mathematics**
**Date**	**25-Aug-2026**
**COI**	**None**

This study aimed to compare the effects of cognitive task-based walking exercise and walking-only exercise on cognitive function, brain fog severity, depressive symptoms, sleep quality, and quality of life in older adults with subjective brain fog.

Reviewer comments:

"Trial Registration: ClinicalTrials.gov, NCT07396779."

and

"The trial was prospectively registered at ClinicalTrials.gov (Identifier: NCT07396779) to ensure methodological transparency"

The authors have suitably registered the trial.

Can the authors please provide a copy of the study protocol and Statistical Analysis Plan (SAP)?

"The study was designed, conducted, and reported in accordance with the Consolidated Standards of Reporting Trials (CONSORT) statement, and the completed CONSORT checklist was provided as supplementary material"

Thanks to the authors for providing a copy of the CONSORT checklist.

"assessor-blinded, parallel-group randomized controlled trial" [Abstract]

and

"This study was conducted as a parallel-group, assessor-blinded randomized controlled trial with a 1:1 allocation ratio. "

The authors have conducted a rigorous study design.

"included 90 community-dwelling adults aged 65 years and older with subjective brain fog symptoms." [Abstract]

and

"The trial was carried out at Istanbul Medipol University Hospital "

and

"As a result, 90 eligible participants were randomized to either the Exercise-Only Group (EX) or the Cognitive Motor Exercise Group (CM-EX), with 45 participants allocated to each group."

Can the authors please assess whether the included cohort can be considered to be representative?

"All randomized participants completed the 4-week follow-up and were included in the final analyses (Figure 1)."

To clarify, are the ITT and PP populations the same?

"Participants were randomly allocated to either a walking-only exercise group (EX; n = 45) or a cognitive–motor exercise group (CM-EX; n = 45)"

and

"Participants who met the eligibility criteria and provided written informed consent were randomly assigned in a 1:1 ratio to either the EX or the CM-EX group."

Can the authors please confirm if there were any stratification variables?

"An a priori power analysis was conducted using G*Power (version 3.1) for the primary outcome, global cognitive function assessed by the MoCA. The analysis was based on an ANCOVA model for a baseline-adjusted between-group comparison, with two groups and one covariate (baseline MoCA score). A standardized effect size of f=0.30 was conservatively assumed for the primary comparison. With a two-tailed α level of 0.05 and 80% power (1-285 β=0.80), the required total sample size was estimated as 90 participants. Although the planned recruitment target was increased to 100 participants to account for possible attrition or incomplete data, 90 participants were ultimately randomized and all completed the study. This final sample therefore met the minimum sample size requirement defined by the a priori power analysis. "

The authors have communicated the basis for the sample size calculation and associated assessment of study power.

"The normality of continuous variables was assessed using the Shapiro Wilk test. Based on the results of the normality assessments, parametric tests were applied for outcomes that met the assumption of normality, whereas non-parametric methods were used for variables that violated this assumption. Descriptive statistics are presented as mean ± SD or median (IQR) for continuous variables, depending on distributional characteristics, and as frequency (percentage) for categorical variables. Baseline demographic and clinical 297 characteristics were compared between groups using the Mann-Whitney U test for continuous variables and the chi-square test or Fisher's exact test, as appropriate, for categorical variables. For cognitive and psychosocial outcomes (MoCA, Brain Fog Scale, and GDS) and quality-of life outcomes measured by the WHOQOL-OLD, within-group changes were analyzed using paired samples t-tests, and unadjusted between-group comparisons at baseline and post intervention were performed using independent samples t-tests. To evaluate post-intervention group differences while adjusting for baseline values, analysis of covariance (ANCOVA) was conducted. Before conducting ANCOVA, the assumptions of linearity, homogeneity of regression slopes, normality of residuals, and homoscedasticity were checked and found to be satisfactory. ANCOVA was selected as the primary between-group analytical approach because it controls for baseline variability and improves statistical precision. Effect sizes for ANCOVA were reported as partial eta squared (η^2p).

Sleep-related outcomes assessed with the PSQI were analyzed using non-parametric methods. Within-group changes were examined using the Wilcoxon signed-rank test, and between-group comparisons were performed using the Mann-Whitney U test."

The authors have applied appropriate statistical tests for the type of data in hand.

"Table 1 Baseline demographic and clinical characteristics of participants across intervention groups."

Can race and ethnicity please be reported here?

Can the authors please clarify how they handled any missing data in the analysis?

The statistical outcomes are reported clearly and accurately in the Results section.

"Figure 1. Flow diagram of the study design."

Can the authors please confirm whether this flowchart follows the CONSORT template?

"The intervention period was limited to four weeks and no long-term follow-up was conducted; therefore, the sustainability of the observed effects remains unclear." [Strengths and Limitations of this study]

and

"This study has several limitations. First, the findings may not be generalisable to older adults with different clinical, cultural or functional profiles. Second, the intervention lasted only four weeks and no long-term follow-up was conducted; therefore, the sustainability of the observed effects remains unknown. Third, although global cognition was assessed using the MoCA, detailed neuropsychological tests targeting attention, executive function, processing speed and working memory were not included. As a result, domain-specific cognitive changes may not have been fully captured. Finally, some secondary outcomes were exploratory, and no formal adjustment was applied for multiple comparisons. Future multicentre trials with longer, progressive intervention protocols, domain-specific cognitive assessments and follow-up evaluations are needed to clarify the added value of cognitive–motor dual-task training in older adults with brain fog"

The authors have provided a Discussion on the study limitations.

VERSION 1 - AUTHOR RESPONSE

Reviewer 1,

Please explain how the exercise intensity was determined, individualised, and monitored during the intervention.

Response:

Thank you for this valuable comment. We have clarified how the prescribed low exercise intensity was determined, individualized and monitored. Exercise intensity was guided by the Borg Rating of Perceived Exertion (RPE) scale (6–20), with a target range of 9–11, corresponding to light exertion. Participants were regularly asked to rate their perceived exertion during sessions. The physiotherapist used these ratings, together with observed

fatigue, gait and balance performance, and overall exercise tolerance, to individualize walking pace and provide brief rest periods when necessary.

Please clarify how the difficulty of the cognitive tasks was standardised and progressed across participants. Was cognitive-task performance formally recorded and used to guide progression, or were adjustments based solely on the physiotherapist's clinical judgement?

Response:

Thank you for this important comment. We have clarified that the cognitive task pool and the type of cognitive demand paired with each walking exercise were standardized across participants. Cognitive-task performance was not formally scored or recorded using a separate cognitive performance measure. Task difficulty and progression were determined based on the supervising physiotherapist's clinical judgement, considering the participant's cognitive performance, gait, postural stability, and safety.

Please provide further information regarding intervention adherence. Did all participants complete every planned exercise session?

Response:

Thank you for this comment. The intervention comprised 12 prescribed sessions, and missed sessions were rescheduled to support programme completion. All participants completed the intervention according to the protocol. Minor adjustments to exercise pace, task difficulty, assistive device use or rest periods were permitted when clinically necessary to accommodate individual exercise tolerance and ensure safety.

Most secondary outcomes were based on self-reported measures. Please acknowledge in the limitations that these outcomes may have been influenced by participants' expectations and subjective reporting.

Response:

Thank you for this valuable suggestion. We have added this issue to the limitations section. We now acknowledge that self-reported outcomes may be influenced by participants' expectations, perceptions of treatment benefit, and subjective response tendencies, and we suggest that future studies combine self-reported measures with more objective behavioral, cognitive, or physiological measures.

Reviewer 2,

Lines 26–27: The title should define the study population more precisely, as participants were specifically older adults with subjective brain fog. Please also consider replacing "cognitive task-based" with the more established term "cognitive–motor dual-task."

Response:

Thank you for this helpful suggestion. We have revised the title to more precisely define the study population as older adults with subjective brain fog and have adopted the more established term "cognitive–motor dual-task" throughout the manuscript. Accordingly, the title has been revised to "Effects of Cognitive-Motor Dual-Task Walking Exercise Versus Walking-Only Exercise on Cognitive Function and Patient-Centered Outcomes in Older Adults with Subjective Brain Fog: A Randomized Controlled Trial." We also replaced the term "cognitive task-based" with "cognitive–motor dual-task" in the short title, Abstract, Introduction, and Conclusion to ensure terminological consistency throughout the manuscript.

Lines 50–52: Brain fog severity is a secondary outcome, baseline scores differed between groups, and no adjustment for multiple comparisons was applied. Please clearly describe this finding as exploratory rather than confirmatory.

Response:

Thank you for this important comment. We have revised the Abstract, Results and Discussion to explicitly describe the between-group finding for brain fog severity as exploratory rather than confirmatory. We now emphasize that brain fog severity was a secondary outcome, baseline scores differed between groups, and no adjustment for multiple comparisons was applied. We have also clarified this issue in the limitations section and avoided interpreting this finding as confirmatory evidence of superiority.

Lines 69–80: This section acknowledges the short intervention period and the lack of follow-up assessment. However, the absence of a non-exercise or usual-care control group should also be reported as an important limitation.

Response:

Thank you for this comment. We have explicitly added the absence of a non-exercise or usual-care control group as an important limitation in the "Strengths and limitations of this study" section. This limitation is also addressed in the Discussion, noting that the observed within-group changes cannot be attributed exclusively to the exercise interventions.

Lines 140–148: The manuscript states that participants were required to have moderate or greater symptom severity according to the Turkish Brain Fog Scale; however, the exact cut-off score is not reported. Please specify the operational cut-off and its supporting reference. If no validated cut-off exists, revise the wording and clarify that the scale was used to quantify symptom severity rather than classify participants into severity categories.

Response:

Thank you for this important comment. We reviewed the original validation study of the Turkish Brain Fog Scale and confirmed that no validated numerical cut-off or established severity categories have been reported. Therefore, the scale was used to quantify symptom severity rather than to classify participants into severity categories, and no numerical cut-off was applied for eligibility. We have clarified this point in the Methods section.

Lines 157–164: It is unclear whether the withdrawals occurred before or after randomization. Please clarify the participant flow, especially since all randomized participants completed follow-up.

Response:

Thank you for this comment. We have clarified that all exclusions occurred before randomization and that all 90 randomized participants completed the week-4 follow-up and were included in the final analyses.

Lines 184–193: The exercises are described as low intensity; however, it is unclear how exercise intensity was determined or monitored. Please clarify the method used.

Response:

Thank you for this comment. We have clarified that the prescribed low exercise intensity was guided by the Borg Rating of Perceived Exertion (RPE) scale (6–20), with a target range of 9–11, corresponding to light exertion. Intensity was monitored and adjusted by the

physiotherapist based on perceived exertion, fatigue, gait and balance performance, and overall exercise tolerance.

Lines 326–327: The absence of statistically significant baseline differences does not establish group comparability. Please revise this statement and note that baseline Brain Fog Scale scores differed between groups.

Response:

Thank you for this comment. We have revised the statement to describe baseline characteristics without implying group comparability and explicitly noted that baseline Brain Fog Scale scores were higher in the CM-EX group than in the EX group.

Lines 349–358: Although the 1–2 point increase in MoCA scores may be clinically meaningful, repeated administration of the same test within four weeks may have introduced a practice effect. Please acknowledge this possibility in the discussion.

Response:

Thank you for this valuable comment. We have acknowledged in the Discussion that repeated administration of the same MoCA within four weeks may have introduced a practice effect and contributed to the observed within-group improvements.

Lines 483–486: Referring to walking-only exercise as a "pragmatic first-line strategy" may overstate the findings, as usual care, feasibility, and cost-effectiveness were not assessed. Please use more cautious wording.

Response:

Thank you for this comment. We have revised the statement to use more cautious wording and have clarified that the role of walking-only exercise relative to usual care, as well as its feasibility and cost-effectiveness, requires further evaluation.

Lines 499–506: The terms "well-tolerated" and "low-cost" are not sufficiently supported by adherence, adverse-event, or cost data. Please use more cautious wording and avoid causal conclusions in the absence of a non-exercise control group.

Response:

Thank you for this comment. We have removed the terms "well-tolerated," "feasible," and "low-cost" and revised the Conclusion to avoid causal interpretations, particularly given the absence of a non-exercise control group.

Lines 514–516: The data availability statement does not specify which data will be shared, the availability period, or the conditions for access. Please provide these details and ensure consistency with the trial registration.

Response:

Thank you for this comment. We have revised the Data Availability Statement to specify the data to be shared, the conditions for access, and the availability period. De-identified participant-level data and the data dictionary will be available for five years from publication upon approval of a scientifically sound proposal and execution of a data-sharing agreement.

Reviewer 3,

Prof. Zoe Kelson, University of Exeter

Comments to the Author:

This study aimed to compare the effects of cognitive task-based walking exercise and walking-only exercise on cognitive function, brain fog severity, depressive symptoms, sleep quality, and quality of life in older adults with subjective brain fog.

Reviewer comments:

"Trial Registration: ClinicalTrials.gov, NCT07396779."

and

"The trial was prospectively registered at ClinicalTrials.gov (Identifier: NCT07396779) to ensure methodological transparency"

The authors have suitably registered the trial.

Response: We thank the reviewer for acknowledging the trial registration.

Can the authors please provide a copy of the study protocol and Statistical Analysis Plan (SAP)?

Response: Thank you for this request. We have provided the study protocol, including the Statistical Analysis Plan (SAP), as a supplementary file entitled "Study Protocol and Statistical Analysis Plan". The document describes the study design, eligibility criteria, interventions, outcome assessments, randomization, blinding and statistical methods.

"The study was designed, conducted, and reported in accordance with the Consolidated Standards of Reporting Trials (CONSORT) statement, and the completed CONSORT checklist was provided as supplementary material"

Thanks to the authors for providing a copy of the CONSORT checklist.

"assessor-blinded, parallel-group randomized controlled trial" [Abstract]

and

"This study was conducted as a parallel-group, assessor-blinded randomized controlled trial with a 1:1 allocation ratio. "

The authors have conducted a rigorous study design.

"included 90 community-dwelling adults aged 65 years and older with subjective brain fog symptoms." [Abstract]

and

"The trial was carried out at Istanbul Medipol University Hospital "

and

"As a result, 90 eligible participants were randomized to either the Exercise-Only Group (EX) or the Cognitive Motor Exercise Group (CM-EX), with 45 participants allocated to each group."

Can the authors please assess whether the included cohort can be considered to be representative?

Response: Thank you for this comment. Participants were recruited from outpatient attendees at Istanbul Medipol University Hospital rather than through population-based sampling. The cohort comprised community-dwelling adults aged ≥65 years with subjective brain fog who met the eligibility criteria, including a MoCA score ≥20, independent ambulation with or without an assistive device, and clinical stability. The findings are therefore most applicable to older adults with similar characteristics. Recruitment from a single hospital and these eligibility

criteria limit generalisability to the broader population, particularly individuals with greater cognitive or mobility impairments or unstable clinical conditions. We have clarified the recruitment source in the Methods and expanded the corresponding limitation in the Discussion.

"All randomized participants completed the 4-week follow-up and were included in the final analyses (Figure 1)."
To clarify, are the ITT and PP populations the same?
Response: Thank you for requesting clarification. Yes, the intention-to-treat and per-protocol populations comprised the same 90 participants. All randomized participants completed the intervention according to the protocol and the four-week assessment, with no missing outcome data. All participants were analysed in their assigned groups. We have clarified this in the Statistical Analysis section.

"Participants were randomly allocated to either a walking-only exercise group (EX; n = 45) or a cognitive–motor exercise group (CM-EX; n = 45)"
and
"Participants who met the eligibility criteria and provided written informed consent were randomly assigned in a 1:1 ratio to either the EX or the CM-EX group."
Can the authors please confirm if there were any stratification variables?
Response: Thank you for requesting clarification. No stratification variables were used in the randomization. We have clarified this in the Randomization and Blinding section.

"An a priori power analysis was conducted using G*Power (version 3.1) for the primary outcome, global cognitive function assessed by the MoCA. The analysis was based on an ANCOVA model for a baseline-adjusted between-group comparison, with two groups and one covariate (baseline MoCA score). A standardized effect size of f=0.30 was conservatively assumed for the primary comparison. With a two-tailed α level of 0.05 and 80% power (1-285 β=0.80), the required total sample size was estimated as 90 participants. Although the planned recruitment target was increased to 100 participants to account for possible attrition or incomplete data, 90 participants were ultimately randomized and all completed the study. This final sample therefore met the minimum sample size requirement defined by the a priori power analysis. "

The authors have communicated the basis for the sample size calculation and associated assessment of study power.

Response: Thank you for your positive assessment of our sample size calculation and power analysis.

"The normality of continuous variables was assessed using the Shapiro Wilk test. Based on the results of the normality assessments, parametric tests were applied for outcomes that met the assumption of normality, whereas non-parametric methods were used for variables that violated this assumption. Descriptive statistics are presented as mean ± SD or median (IQR) for continuous variables, depending on distributional characteristics, and as frequency (percentage) for categorical variables. Baseline demographic and clinical 297 characteristics were compared between groups using the Mann-Whitney U test for continuous variables and the chi-square test or Fisher's exact test, as appropriate, for categorical variables. For cognitive and psychosocial outcomes (MoCA, Brain Fog Scale, and GDS) and quality-of life outcomes measured by the WHOQOL-OLD, within-group changes were analyzed using paired samples t-tests, and unadjusted between-group comparisons at baseline and post intervention were performed using independent samples t-tests. To evaluate post-intervention group differences while adjusting for baseline values, analysis of covariance (ANCOVA) was conducted. Before conducting ANCOVA, the assumptions of linearity, homogeneity of regression slopes, normality of residuals, and homoscedasticity were checked and found to be satisfactory. ANCOVA was selected as the primary between-group analytical approach because it controls for baseline variability and improves statistical precision. Effect sizes for ANCOVA were reported as partial eta squared (η^2p).
Sleep-related outcomes assessed with the PSQI were analyzed using non-parametric methods. Within-group changes were examined using the Wilcoxon signed-rank test, and between-group comparisons were performed using the Mann-Whitney U test."
The authors have applied appropriate statistical tests for the type of data in hand.
Response: Thank you for your positive assessment of our statistical methods.

"Table 1 Baseline demographic and clinical characteristics of participants across intervention groups."
Can race and ethnicity please be reported here?

Response: Thank you for this suggestion. Race and ethnicity were not collected in this study and therefore cannot be reported in Table 1.

Can the authors please clarify how they handled any missing data in the analysis?

Response: No outcome data were missing at baseline or week 4; therefore, no imputation was required. We have clarified this in the Statistical Analysis section.

The statistical outcomes are reported clearly and accurately in the Results section.

"Figure 1. Flow diagram of the study design."

Can the authors please confirm whether this flowchart follows the CONSORT template?

Response: Thank you for requesting clarification. Figure 1 follows the CONSORT participant flow structure. We have revised it to explicitly report receipt of the allocated intervention, losses to follow-up, intervention discontinuations and exclusions from analysis for each group.

"The intervention period was limited to four weeks and no long-term follow-up was conducted; therefore, the sustainability of the observed effects remains unclear." [Strengths and Limitations of this study]

and

"This study has several limitations. First, the findings may not be generalisable to older adults with different clinical, cultural or functional profiles. Second, the intervention lasted only four weeks and no long-term follow-up was conducted; therefore, the sustainability of the observed effects remains unknown. Third, although global cognition was assessed using the MoCA, detailed neuropsychological tests targeting attention, executive function, processing speed and working memory were not included. As a result, domain-specific cognitive changes may not have been fully captured. Finally, some secondary outcomes were exploratory, and no formal adjustment was applied for multiple comparisons. Future multicentre trials with longer, progressive intervention protocols, domain-specific cognitive assessments and follow-up evaluations are needed to clarify the added value of cognitive–motor dual-task training in older adults with brain fog"

The authors have provided a Discussion on the study limitations.

Response: Thank you for your positive assessment of our discussion of the study limitations.
