# Supplementary material for: Effects of cognitive-motor dual-task walking exercise versus walking-only exercise on cognitive function and patient-centred outcomes in older adults with subjective brain fog: a randomised controlled trial
Source: BMJ Open. 2026 Oct 20;16(10):e121892. doi: 10.1136/bmjopen-2026-121892 (PMC13684161; doi:10.1136/bmjopen-2026-121892)
Supplement: Supplementary file 2 [file bmjopen-16-10-s001.docx]

|  | **ISTANBUL MEDIPOL UNIVERSITY**  **Study Protocol and Statistical Analysis Plan** |
| --- | --- |

Cognitive–motor walking exercise versus walking-only exercise in older adults with subjective brain fog.

## Study overview

| Characteristic | Specification |
| --- | --- |
| Design | Single-centre, parallel-group, assessor-blinded randomized superiority trial |
| Allocation | 1:1 to walking-only exercise or cognitive–motor walking exercise |
| Setting | Istanbul Medipol University Hospital, Istanbul, Türkiye |
| Investigators | Emre Dansuk and Burak Menek |
| Recruitment target | 100 participants to allow for potential attrition |
| Intervention | Four weeks; three individually supervised sessions weekly |
| Primary endpoint | MoCA total score at the end of week 4 |

## Rationale and objectives

Subjective brain fog encompasses perceived difficulties in attention, concentration and mental clarity. Walking with concurrent cognitive tasks may address the divided attention demands of everyday mobility. This study will determine whether adding cognitive tasks to a standardized walking programme provides greater short-term benefit than walking-only exercise.

The primary objective will be to compare global cognitive function after four weeks. Secondary objectives will be to compare brain fog severity, depressive symptoms, sleep quality and quality of life. We hypothesize that cognitive–motor exercise will improve these outcomes more than walking-only exercise. The trial will test superiority; a non-significant result will not establish equivalence.

## Sample size- G power

The minimum required sample size was calculated as 90 participants using G-Power 3.1 for an ANCOVA F test with two groups, one baseline covariate, and one numerator degree of freedom, assuming an effect size of f = 0.30, α = 0.05, and 80% power. The target sample size will be increased to 100 participants to allow for approximately 10% attrition.

# Participants and study procedures

## Eligibility and screening

Eligible participants will be community-dwelling adults aged ≥65 years who report reduced attention, concentration or mental clarity for at least four weeks before enrolment. Brain fog will be operationalized as a subjective symptom construct rather than a formal clinical diagnosis. Additional eligibility criteria will include a Montreal Cognitive Assessment (MoCA) score ≥20, independent ambulation with or without an assistive device, and no acute illness or medication changes within the preceding four weeks. The Turkish Brain Fog Scale will be used to quantify symptom severity.

A physician will screen participants before allocation. Exclusion criteria will include diagnosed neurodegenerative disease, previous stroke or other major neurological disease, delirium, severe psychiatric disorder, medical contraindications to exercise, severe visual or hearing impairment interfering with participation, and inability to follow study procedures.

**Consent and allocation**

Written informed consent will be obtained before enrolment. An independent researcher who will not be involved in recruitment, intervention delivery or outcome assessment will generate the random allocation sequence using Research Randomizer ([www.randomizer.org](http://www.randomizer.org)). Participants will be allocated in a 1:1 ratio to the EX or CM-EX group. The same researcher will prepare sequentially numbered, opaque, sealed envelopes to conceal allocation. After each participant’s baseline assessment is completed, the treating physiotherapist will open the next envelope in sequence and deliver the allocated intervention.

**Blinding**

Participants and the treating physiotherapist will not be blinded because of the nature of the interventions. All baseline and post-intervention assessments will be performed by an outcome assessor who will not deliver the interventions and will remain blinded to group allocation. Participants will be instructed not to disclose their intervention to the assessor. An independent statistician will analyse data coded as groups A and B, without access to intervention descriptions or the correspondence between group codes and interventions until the primary analyses are completed. The treating physiotherapist will retain this information.

## Assessments and endpoints

Assessments will be performed at baseline and immediately after the four-week programme by the blinded assessor. Each participant will follow the same assessment schedule. No longer-term follow-up is included.

| **Measure** | **Endpoint and interpretation** |
| --- | --- |
| MoCA total | Primary; 0–30; higher indicates better global cognition |
| Brain Fog Scale total | Secondary; 30 items; higher indicates greater symptom severity |
| GDS 30-item total | Secondary; 0–30; higher indicates greater depressive symptoms |
| PSQI global | Secondary; 0–21; higher indicates poorer sleep quality |
| WHOQOL-OLD total | Secondary; higher indicates better quality of life |

# Interventions and trial management

## Shared exercise programme

Both groups will receive individually supervised low-intensity walking exercise from a physiotherapist, three times per week for four weeks, comprising 12 prescribed sessions. Each session will last 35 minutes: a 5-minute warm-up, 25-minute main phase and 5-minute cool-down. Warm-up and cool-down will include marching in place and slow straight-line walking. Pace and support will be individualized according to physical capacity, chronic disease history, ambulatory status and balance. Exercise intensity will be guided by the Borg Rating of Perceived Exertion (RPE) scale (6–20), with a target range of 9–11. Participants will be regularly asked to rate their perceived exertion during sessions. The physiotherapist will use these ratings, together with observed fatigue, gait and balance performance, and overall exercise tolerance, to adjust walking pace or provide brief rest periods when necessary.

| **Motor task** | **Duration** | **Concurrent cognitive task in CM-EX** |
| --- | --- | --- |
| Tandem walking | About 5 minutes | Forward/backward counting or numerical sequences |
| Side walking | About 5 minutes | Letter or semantic-category verbal fluency |
| Figure-of-eight walking | About 5 minutes | Days or months in forward or reverse order |
| Backward walking | About 5 minutes | Backward counting or another simple mental task |
| Walking with a light object | About 5 minutes | Category tasks, simple recall or sequencing |

## Walking-only exercise group

The EX-group will perform the five walking tasks without an added cognitive task. A light object, such as a small ball or a plastic cup containing water, may be carried during the final task. Walking tasks will be delivered in a safe environment with assistive devices or external support when necessary.

## Cognitive–motor exercise group

The CM-EX group will follow the same motor sequence, session duration, frequency and supervision, while completing verbally delivered cognitive tasks during the main walking phase. Tasks will target attention, working memory, verbal fluency and sequencing. Cognitive difficulty will be adjusted to cognitive performance, tolerance and gait safety.

## Safety and adherence

Gait safety and postural stability will take priority over cognitive-task performance. The physiotherapist will monitor balance, fatigue and tolerance. Brief rest, pace adjustment, environmental support, task simplification or temporary suspension of the cognitive task will be permitted when clinically necessary.

During each session, the physiotherapist will monitor participants for adverse events. Any event will be documented, and the exercise will be paused or modified when clinically necessary. Missed sessions will be rescheduled to support completion of the prescribed 12-session programme. Participants will be encouraged to attend the post-intervention outcome assessment regardless of the number of sessions completed.

**Statistical analysis plan**

## Analysis population and conventions

The principal analysis will compare participants according to randomized allocation. IBM SPSS Statistics 22.0 will be used. Tests will be two-sided, with α=0.05 for the primary comparison. Continuous data will be summarized using means and standard deviations or medians and interquartile ranges, and categorical data using counts and percentages. Normality will be assessed using the Shapiro–Wilk test.

**Primary analysis**

Week-4 MoCA total score will be analysed using ANCOVA to compare groups while adjusting for baseline MoCA scores. The statistician will analyse groups coded as A and B and will remain blinded to group identity until the primary analyses are completed. P values and partial eta squared (η²p) will be reported. Linearity, homogeneity of regression slopes, residual normality and homoscedasticity will be assessed.

## Secondary and exploratory analyses

## Brain Fog Scale, GDS and WHOQOL-OLD scores will be analysed using baseline-adjusted ANCOVA. For outcomes suitable for parametric analysis, paired-samples t-tests will assess within-group changes, and independent-samples t-tests will assess unadjusted between-group differences at baseline and week 4. PSQI outcomes will be analysed using Wilcoxon signed-rank tests for within-group changes and Mann–Whitney U tests for between-group comparisons. No adjustment for multiple comparisons will be applied; secondary findings will be interpreted as exploratory.

## Effect sizes

ANCOVA effect size will be partial eta squared, calculated as SSgroup / (SSgroup + SSerror). Wilcoxon effect size will be r=Z/√N.
